# Algorithm Improves Acetabular Fracture Radiograph Interpretation Among Inexperienced Practitioners

**DOI:** 10.7759/cureus.21471

**Published:** 2022-01-21

**Authors:** Bennet A Butler, Ryan S Selley, Colin K Cantrell, Richard W Nicolay, Cort D Lawton, Sohaib Z Hashmi, Kevin R Carlile, Michael D Stover

**Affiliations:** 1 Department of Orthopaedic Surgery, Northwestern University Feinberg School of Medicine, Chicago, USA; 2 Orthopaedic Surgery, Ortho Illinois, Chicago, USA

**Keywords:** pelvic acetabulum fractures, acetabulum, acetabular fracture, classification, education

## Abstract

Acetabular fractures are often first evaluated in the emergency department, where physicians with little experience reading pelvic radiographs may be required to make an accurate diagnosis and early management decisions. In this study, medical students classified radiographs of 20 acetabular fractures and repeated the exercise three weeks later with the aid of a previously described algorithm; half the students were given a lesson prior to using the algorithm. The pre-algorithm accuracy was 4/20 and the post-algorithm accuracy was 8.3/20 (p<0.01). The lesson provided no difference (p=0.5). This algorithm is therefore a useful reference to help classify and triage acetabular fractures.

## Introduction

Acetabular fractures are serious injuries generally caused by high energy blunt force trauma, although increasingly there is a subset of lower energy injuries observed in elderly populations [1]. The Judet and Letournel acetabular classification system was developed to help surgeons properly describe these fractures and plan their surgical approach [2, 3]. Differentiating acetabular fracture types is also important in the acute setting, as certain patterns are associated with significant blood loss or may need skeletal traction to prevent additional joint damage [4].

Experienced surgeons are able to classify fractures based on the Letournel system with high intra- and interobserver reliability [5]. For less experienced attending surgeons and residents, however, properly classifying acetabular fractures can be difficult [6]. Efforts to develop teaching strategies aimed at these practitioners, frequently involving advanced imaging such as CT scan with 2D and 3D reconstructions, have met with mixed results [6-9].

Some authors have described algorithms that can be used to help less experienced readers properly classify acetabular fractures. These have been shown to be effective teaching tools primarily for attending surgeons and senior residents [10]. An algorithm developed by Saterbak et al. [11] and modified by Ly et al. [12] was shown to improve the ability of all residents, including interns, to accurately use the Judet and Letournel classification system.

Practically, however, these fractures are often first encountered in the emergency department, where the burden of diagnosis may be placed on physicians with little to no experience reading radiographs of acetabular fractures. These include resident radiologists, general trauma surgeons, emergency medicine doctors and, early in the academic year, new orthopedic interns. A method of quickly teaching these practitioners to accurately diagnose acetabular fractures and communicate that diagnosis with other health care providers may help to direct initial care and final patient disposition.

This study aims to assess the effectiveness of a previously developed algorithm for teaching inexperienced practitioners to accurately classify acetabular fractures using the Judet and Letournel system.

## Materials and methods

Pre-test

**Figure 1:**
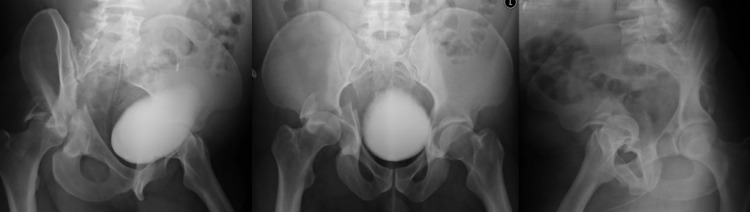
Obturator oblique, anteroposterior, and iliac oblique radiographs of an acetabular fracture

This study was approved by our Institutional Review Board. Fourth-year medical students on their orthopedic surgery rotation were recruited for the study. They were instructed that there would be a series of tests but were not informed of the topic or that the tests would be on the same topic. They were explicitly instructed to not discuss nor utilize independent study before, between or after each testing session. Each student was given a test composed of the radiographs (anteroposterior (AP), obturator oblique, and iliac oblique view) of 20 separate acetabular fractures (Figure 1).

These fractures were classified previously by an attending orthopedic trauma surgeon with significant experience treating acetabular fractures. For each set of radiographs, they were instructed to classify the fracture based on the Judet and Letournel classification system [3]. They were informed that patterns may be repeated or excluded altogether.

Post-test

**Figure 2:**
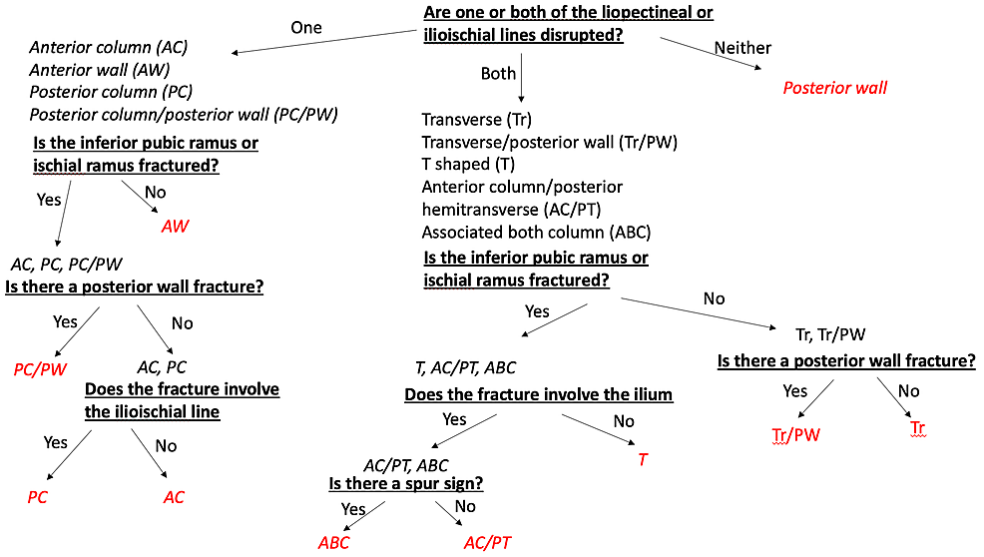
Algorithm developed by Ly et al. and provided to medical students for use during their post-test Ly et al. [12]

After a 3-week washout period, the students once again took the same test that they had taken previously. This time, however, they were provided with the algorithm previously described by Ly et al. [12] (Figure 2).

Half of the students were given a short presentation explaining the algorithm and its proper use. Otherwise, the testing protocol was identical to that of the pre-test. 

Test validation

The test taken by the medical students was validated using resident scores on the same test. Residents ranging from PGY1 to PGY5 were given the test without any specific preparation. The residents were provided with the same instructions as the medical students. Their scores were compared to determine if the test could differentiate residents with varying levels of training.

Statistics

Test scores were collected and reported as means with standard deviations. Student’s t-tests were performed as appropriate to compare groups. A p-value of <0.05 (two-tailed) was considered statistically significant. Analyses were generated using JMP version 9.0.2 (SAS Institutes, Inc., Cary, USA).

## Results

Testing

A total of 25 medical students took part in the study. On their pre-test, without the help of the algorithm, medical students scored an average of 4.04 questions correct out of 20. On their post-test, with the help of the algorithm, medical students scored an average of 8.32 questions correct (p<0.01). The medical students significantly improved their scores with respect to both elementary (p<0.01) and associated (p<0.01) fracture patterns (Table 1).

**Table 1:** Medical student pre- and post-test scores (with standard deviations)

	Pre-Test (n=25)	Post-Test (n=25)	P-value
All Fractures	4.04 (3.37)	8.32 (2.98)	<0.001
Elementary Fractures	1.84 (1.49)	3.56 (1.15)	<0.001
Associated Fractures	2.2 (2.17)	4.76 (2.38)	<0.001

Twelve students were randomly separated and given a short presentation explaining the algorithm and its use. This group performed no better than the group of students provided with the algorithm alone (p=0.5) (Table 2).

**Table 2:** Medical student score with and without the addition of short lesson (with standard deviations)

	Pre-Test	Post-Test	
Lesson and Algorithm (n=12)	4.33 (2.77)	8.75 (2.3)	
Algorithm Only (n=13)	3.76 (3.94)	7.92 (3.54)	
P-Value	0.67	0.5	

Test validation

Thirty-nine residents ranging from PGY1 to PGY5 were given the test without specific preparation. Junior residents (PGY1-3) performed significantly better than medical students prior to intervention (p<0.01); senior residents (PGY4-5) performed significantly better than junior residents (p=0.048). The test was, therefore, able to differentiate subjects with varying levels of training (Table 3). 

**Table 3:** Test validation (pre-intervention scores)

Comparison	Difference	P-Value
Medical Student vs Junior Resident	6.03	<0.01
Junior Resident vs Senior Resident	2.09	0.048

## Discussion

Acetabular fractures are complex injuries that can be difficult to appropriately classify and triage [1]. The most common classification system is that of Judet and Letournel, which separates fractures into the 10 most common patterns, five elementary and five associated patterns [2, 3]. In general, this classification system has been found to reliably predict approach and fixation strategy for a wide variety of fracture patterns, although Letournel accepted that transitional patterns exist [13].

One major advantage of the Letournel classification system is that it relies on plain radiographs alone, specifically the anteroposterior, iliac oblique, and obturator oblique views. This is useful given the wide availability of radiographs in most, if not all hospital settings and acuities.

For experienced surgeons, these images allow accurate classification of acetabular fractures with high intra- and interobserver reliability, likely due to holistic pattern recognition [5, 14]. For less experienced surgeons and residents, however, classifying acetabular fractures can be a difficult task. For example, Polesello et al. reported that residents were only able to classify fractures correctly 23% of the time [6]. For this reason, there have been efforts to develop teaching aids to help these less experienced practitioners [6-9].

At the most basic level, multiple authors have shown that providing residents and attending surgeons with advanced imaging, including 2D and 3D CT scans, can improve their diagnostic abilities [7, 15]. Techniques such as secondary image manipulation and 3D printing of CT scan models have also been described to assist residents [8-9].

In parallel to these efforts, other authors have attempted to improve junior attending and resident acetabular fracture classification without resorting to advanced imaging techniques, which may or may not be available in every setting. These authors have tended to rely on algorithms that ask practitioners to assess imaging in a systematic manner rather than holistically. Based on the specific features observed in each step of the algorithm, a final diagnosis can be made.

Patel et al. [10], using an algorithm developed by Brandser and Prevezas demonstrated that their algorithms were effective when given to attending surgeons. Ly et al. [12], using a modified algorithm originally developed by Saterbak et al. [11], showed that their algorithm was effective when given to residents.

Our study expands on the work of Ly et al. and demonstrates that the algorithm described in their study is effective even for practitioners with little to no experience reading acetabular fracture films. This is important because frequently, orthopedic surgeons are not the first physicians tasked with diagnosing and triaging an acute acetabular fracture. Often, patients with these injuries present to emergency departments, where their pelvic radiographs are read by general trauma surgeons, emergency medicine doctors or radiologists with variable musculoskeletal experience. For these practitioners, the algorithm described above can serve as a valuable reference and can help them correctly classify acetabular fractures, appropriately triage them and accurately describe their patients’ injuries to other health care providers. Additionally, new orthopedic interns would likely find this algorithm to be a useful introduction to acetabular fracture classification; it might therefore serve as a valuable addition to intern onboarding curricula and a foundation for future learning.

It was interesting to note that the addition of a short lesson explaining the algorithm did not help subjects with fracture classification. This result may seem unexpected, but it is consistent with the relative simplicity of the algorithm. The algorithm, with few exceptions, asks readers to identify fracture lines through specific anatomic structures; it is, therefore, possible that any reader with a basic understanding of pelvic anatomy could appropriately apply the algorithm without specific coaching. 

This study has a number of weaknesses. For one, there is no guarantee that subjects did not study acetabular fracture classification between their pre and post-tests, in spite of specific instruction not to. Additionally, medical students are not perfect surrogates for the practitioners noted earlier in this discussion. Finally, further studies would be needed to determine if the adoption of the algorithm improves communication between non-orthopedic practitioners and orthopedic surgeons, and ultimately if that improved communication leads to better patient outcomes.

## Conclusions

Inexperienced practitioners were better able to interpret acetabular fracture radiographs with the help of an algorithm. This algorithm can therefore serve as a reference for inexperienced practitioners and can help them correctly classify and triage acetabular fractures.
